# Genetic Diversity and Phylogenetic Relatedness of *Plasmodium ovale curtisi* and *Plasmodium ovale wallikeri* in sub-Saharan Africa

**DOI:** 10.3390/microorganisms10061147

**Published:** 2022-06-02

**Authors:** Mary A. Oboh, Bolaji N. Thomas

**Affiliations:** 1 Department of Biomedical Sciences, Rochester Institute of Technology, Rochester, NY 14623, USA; maochst@rit.edu; 2 Malaria Genomic Epidemiology Network Nigeria—MGENN, Nigeria Institute of Medical Research, Lagos 101245, Nigeria

**Keywords:** *Plasmodium ovale curtisi*, *Plasmodium ovale wallikeri*, genetic diversity, phylogenetic relatedness, sub-Saharan Africa

## Abstract

*P. ovale* was until recently thought to be a single unique species. However, the deployment of more sensitive tools has led to increased diagnostic sensitivity, including new evidence supporting the presence of two sympatric species: *P. ovale curtisi* (Poc) and *P. ovale wallikeri* (Pow). The increased reports and evolution of *P. ovale* subspecies are concerning for sub-Saharan Africa where the greatest burden of malaria is borne. Employing published sequence data, we set out to decipher the genetic diversity and phylogenetic relatedness of *P. ovale curtisi* and *P. ovale wallikeri* using the tryptophan-rich protein and small subunit ribosomal RNA genes from Gabon, Senegal, Ethiopia and Kenya. Higher number of segregating sites were recorded in Poc isolates from Gabon than from Ethiopia, with a similar trend in the number of haplotypes. With regards to Pow, the number of segregating sites and haplotypes from Ethiopia were higher than from those in Gabon. Poc from Kenya, had higher segregating sites (20), and haplotypes (4) than isolates from Senegal (8 and 3 respectively), while nucleotide from Senegal were more diverse (θw = 0.02159; π = 0.02159) than those from Kenya (θw = 0.01452; π = 0.01583). Phylogenetic tree construction reveal two large clades with Poc from Gabon and Ethiopia, and distinct Gabonese and Ethiopian clades on opposite ends. A similar observation was recorded for the phylogeny of Poc isolates from Kenya and Senegal. With such results, there is a high potential that ovale malaria control measures deployed in one country may be effective in the other since parasite from both countries show some degree of relatedness. How this translates to malaria control efforts throughout the continent would be next step deserving more studies.

## 1. Introduction

Malaria resulting from infection with *Plasmodium ovale* (Po) species is difficult to diagnose using conventional light microscopy and/or rapid diagnostic test kit [1] due to several reasons. Such include the low parasite density, potentially mediated by the predisposition of *Po* to reticulocytes, morphological similarity to *P. vivax*, efficient hypnozoitic dormancy resulting in relapse months and years later [2,3], as well as co-infection with *P. falciparum* and *P. malariae*, particular in highly endemic areas with substantial immune response in local populations [4]. As a result, the true burden of Po infection in sub-Saharan Africa, capped by the World Health Organization to range between 1–2% [5] is believed to be grossly underestimated. For instance, employing PCR method for disease diagnosis revealed a prevalence of ≥14% in southwestern Nigeria, which in comparison to microscopy gave an estimate of ≤5% [6,7,8].

*P. ovale* was until recently thought to be a single unique species. However, the deployment of more sensitive tools [9,10] has led to increased diagnostic sensitivity, including new evidence supporting the presence of two sympatric subspecies: *P. ovale curtisi* (Poc) and *P. ovale wallikeri* (Pow) in circulating field isolates [11,12]. These two subspecies co-exist within the same geographical area, sometimes co-infecting same individual [13,14], and are genetically distinct [3]. *P. ovale* infection is considered benign due to minimal investigative research effort to study its biology as a result of difficulty in establishing *in vitro* laboratory cultures [7]. In addition, there is paucity of data on the transmission burden, geographical distribution as well as subspecific diversity. 

The increased reports from Asia on the evolution of *P. ovale* [15,16], severe malaria resulting from *P. ovale* [17] and Poc infections [18], association of *P. ovale* with splenic rupture [19], variation in latency duration observed in both Poc and Pow [14], association of relapse with Poc [20], possibility of re-introduction of other species of malaria after the elimination of the major *P. falciparum* and/or *P. vivax* species as has been reported in Sri Lanka [21,22], are concerning for sub-Saharan Africa. Moreover, a recent study reported the occurrence of Pow in gorillas from central Africa, probably a zoonotic transmission [23]. Therefore, elucidating the intra- and inter-population diversity of this parasite, within and between asymptomatic and clinical disease groups, in sub-Saharan Africa, deserves urgent deconvolution.

Clearly, the public health impact of *P. ovale* as with the subspecies is largely underestimated. With malaria elimination target set for 2030 in many of the endemic countries, it becomes imperative to understand the diversity and geographical spread of *P. ovale* species on the continent that bears the highest malaria burden. To this end, we performed a comparative genomic analysis to decipher the genetic diversity and phylogenetic relatedness of *P. ovale curtisi* and *P. ovale wallikeri* from Kenya (representing East Africa) and Senegal (representing West Africa) using the tryptophan-rich protein gene, as well as from Gabon (representing Central Africa) and Ethiopia (representing Horn of Africa) using the small subunit ribosomal RNA.

## 2. Materials and Methods

A literature search of four research databases was conducted in PubMed, Scopus, Web of Science and Google Scholar. The search terms include “*Plasmodium ovale* species diversity in Africa”; “Diversity of *Plasmodium ovale* species” and “Africa”; “*Plasmodium ovale curtisi* and *Plasmodium ovale wallikeri* diversity” and “Africa”; “*Plasmodium ovale curtisi* and *Plasmodium ovale wallikeri* diversity in Africa”, “*Plasmodium ovale* or *Plasmodium ovale curtisi* or *Plasmodium ovale wallikeri* and Africa”.

### 2.1. Eligibility Criteria of Included Studies

All types of primary studies reporting the occurrence of *P. ovale curtisi* and/or *P. ovale wallikeri* from different African countries with sequence result of any genes from any of these subspecies were included in the analysis. Specifically, and for comparative purposes, we retrieved sequences of the same gene of each subspecies from studies with sequences. Studies with sequences of genes in any of the subspecies reported from individuals who visited any country in Africa briefly were excluded since *P. ovale* undergoes liver dormant stage with possibility for relapse weeks and months later, hence infection cannot be definitively tied to Africa. Furthermore, studies from Africa that do not specifically state the countries where the samples were collected from were excluded. In addition, review studies were also excluded.

### 2.2. Sequences

**Figure 1:**
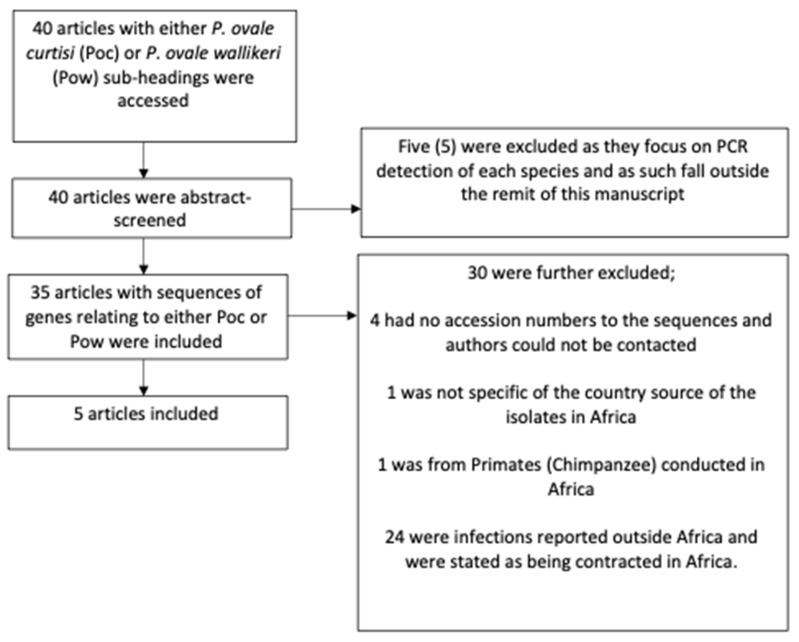
Flowchart of data retrieval and analysis process.

Nucleotide sequences of genes from each species were retrieved from the National Center for Biotechnology Information (NCBI) database (https://www.ncbi.nlm.nih.gov), last accessed on 15 May 2022. A flowchart of the data retrieval and analysis process, including exclusion and inclusion criteria for studies selected for analysis is as shown (Figure 1). A summary of the number of sequences retrieved from the different countries and selected target genes is provided (Table 1).

### 2.3. Sequence Analyses

Since the principal idea behind this work is comparative genetics, only genes studied in the two subspecies across different countries were analyzed. Population genetic analyses were carried out on each of these genes/per subspecies per country to probe the genetic diversity and population structure. First, the haplotype and nucleotide diversities (as estimated by θw and π) for each country and subspecies were independently estimated for each gene [27]. Secondly, the average number of pair-wise nucleotide difference per site was estimated by π [28], and the θw value was based on the number of segregating sites in a population [29], followed by the average number of nucleotide differences between the sequences. All the aforementioned diversity indices provide a basis for evaluating how similar or diverse sequences/parasite isolates in this case are from each other. Thirdly, the neutral model of molecular evolution was assessed by calculation of Tajima’s D (TD), performed for each gene separately for the different countries. The TD test calculates the normalized difference between θw and π. A negative value indicates an excess of low frequency polymorphism, specifying directional selection or population size expansion, while a positive value demonstrates balancing selection or population size reduction. The above evolutionary analyses were carried out using the DnaSP computer program version 5.0 [30].

Finally, to evaluate the phylogenetic relatedness of Poc and Pow isolates between countries, a phylogenetic tree was reconstructed using Neighbor-Joining (NJ) method with 1000 bootstrap replications [31].

## 3. Results

On the basis of our inclusion criteria (Figure 1), we retrieved 38 sequences of different genes from five countries viz Gabon, Ethiopia, Kenya, Senegal and Central Africa Republic (Table 1). For Gabon, sequences from small subunit ribosomal RNA (*SSU rRNA*) gene for Poc (*n* = 12) and Pow (*n* = 5) were retrieved. For Ethiopia; small subunit ribosomal RNA gene for Poc (*n* = 2) and Pow (*n* = 2) were retrieved. For Kenya, sequences retrieved were those of tryptophan-rich protein (tra) with 4 sequences retrieved for Poc and 5 for Pow. Similarly, the tra gene for Poc (*n* = 3) and Pow (*n* = 2) were obtained for Senegalese isolates. For Central African Republic, the cytochrome oxidase subunit 1 of Pow (*n* = 3) was the only sequence retrieved (Table 1). To this end, the genetic population analysis was based on countries with similar genes in each subspecies. For instance, Poc from Gabon and Ethiopia were compared because they both had the *SSU rRNA* gene sequences. Since there was no other country reporting cytochrome oxidase subunit 1 sequence, and that the sequence obtained is only for Pow, Central African Republic was excluded from further analyses.

### 3.1. Genetic Diversity of Small Subunit Ribosomal RNA (SSU rRNA) Gene of Poc and Pow from Gabon and Ethiopia

As per the *SSU rRNA* gene, the number of segregating sites among Gabonese isolates was six; no segregating site in the Poc from Ethiopia was observed, demonstrating that there was no diversity between Ethiopian Poc isolates. Similar trend was observed when the number of haplotypes between both countries were compared, with Gabon having 5 haplotypes and 1 haplotype from Ethiopia, hence, a high haplotype diversity (0.667) and moderate nucleotide diversity (θw = 0.230; π = 0.00359), unlike in Gabon where both haplotype and nucleotide diversities were nil.

However, with regards to Pow between these two countries, a reverse result was observed. The number of segregating sites in Pow from Ethiopia was 3 while there was no observable segregating site from Gabon. Moreover, the number of haplotypes for the former was 2 while only 1 haplotype was noted in the latter. Haplotype and nucleotide diversities were also noted to be high in Ethiopia compared to Gabon (Table 2).

### 3.2. Genetic Diversity of Tryptophan-Rich Antigen (tra) of Poc and Pow from Kenya and Senegal

The number of segregating sites in Kenya isolates (20) was more than double that observed in Senegal (8). In terms of the number of haplotypes, four were observed in Kenya and three in Senegal. Same haplotype diversity was observed between both countries for Poc, but the nucleotide diversity of isolates from Senegal was higher (θw = 0.02159; π = 0.02159) than that observed in Kenya (θw = 0.01452; π = 0.01583).

For Pow, the number of segregating sites observed in Kenya (6) was 1.5 times higher than what was seen in Senegal (4), while the number of haplotypes in Kenya (5) was more than twice that obtained from Senegal (2) (Table 3). However, as in the Poc, the haplotype diversity was similar between both countries. Interestingly, the nucleotide diversity was higher in Senegal (θw = 0.01980; π = 0.01980) as compared to Kenya (θw = 0.00455; π = 0.00468) (Table 3). 

Comparative evolutionary pattern between countries could not be carried out because sequences from some countries were less than four. Per the requirement of the DnaSP program, Version 5.0 [30], such analysis requires more than four sequences.

### 3.3. Phylogenetic Relatedness of Poc and Pow among Countries

**Figure 2:**
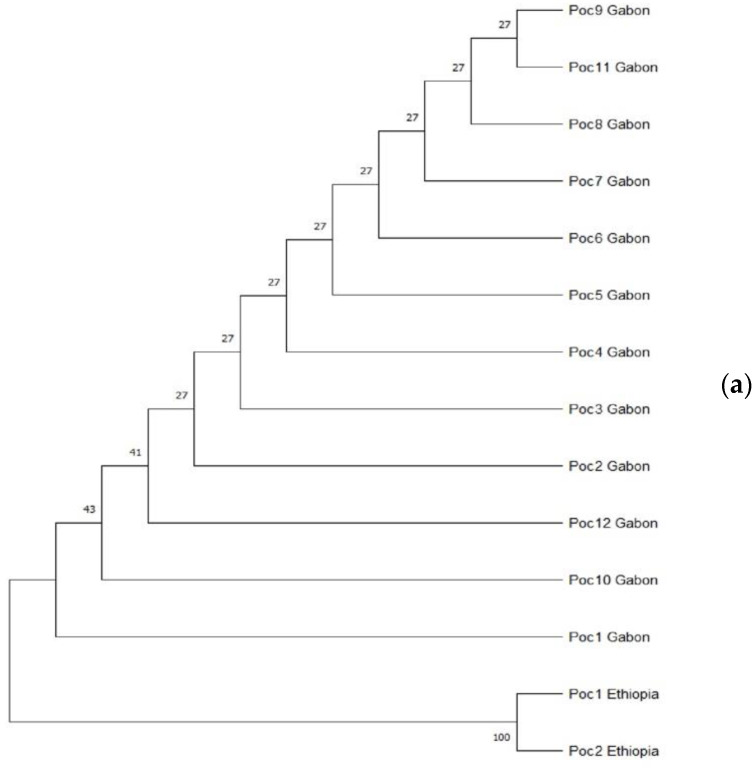

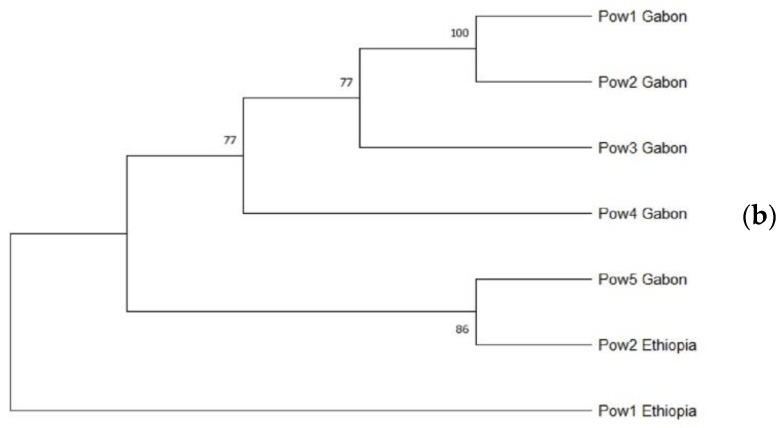
Phylogenetic profile of *Plasmodium ovale* subspecies from different parts of Africa; profiling with small subunit ribosomal RNA gene of *P. ovale curtisi* from Gabon and Ethiopia (**a**); profiling with small subunit ribosomal RNA gene of *P. ovale wallikeri* from Gabon and Ethiopia (**b**).

The Neighbor-Joining phylogenetic tree reconstructed with 1000 bootstrap iterations for the *SSU rRNA* gene in the Poc from Gabon and Ethiopia revealed two large clades with all the isolates from Gabon on one side and those from Ethiopia forming a separate clade, depicting low similarity (Figure 2a). On the other hand, the reconstructed tree of Pow between both countries showed two clades, with an Ethiopian isolate (Pow2_Ethiopia) forming a separate clade with a Gabonese isolate (Pow5_Gabon) and a bootstrap value of 86, depicting very high similarity (Figure 2b).

**Figure 3:**
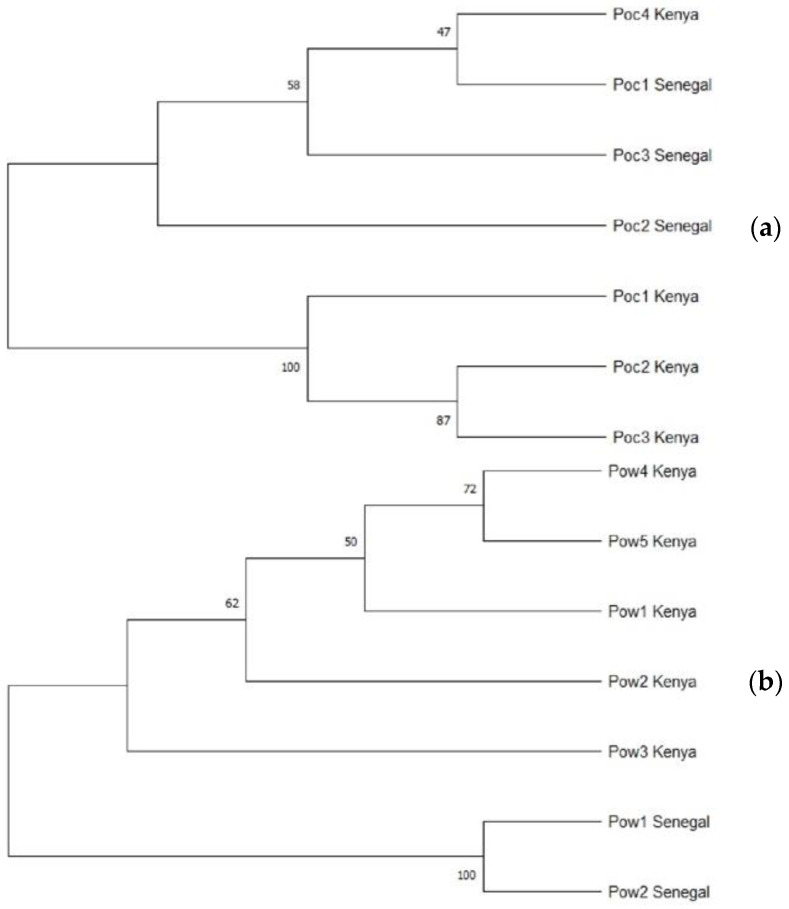
Phylogenetic profile of *Plasmodium ovale* subspecies from different parts of Africa; profiling with tryptophan-rich antigen gene of *P. ovale curtisi* from Kenya and Senegal (**a**); profiling with tryptophan-rich antigen gene of *P. ovale wallikeri* from Kenya and Senegal (**b**).

Similarly, the reconstructed phylogeny of Poc isolates from Kenya and Senegal demonstrated with the tra gene showed the formation of a unique clade between an isolate from Senegal (Poc1_Senegal) and another from Kenya (Poc4_Kenya) (Figure 3a), while the other Kenya isolates formed a separate clade with themselves. With regards to the Pow isolates, there was a distinct clade formation among isolates from each country (Figure 3b).

## 4. Discussion

Until recently, there was little interest on the prevalence, geographical spread and diversity of *P. ovale curtisi* and *P. ovale wallikeri*. With renewed interest in malaria elimination [32], and much attention given to non-*P. falciparum* malaria and their role in disease transmission [33] and the re-introduction of malaria in previously eliminated regions [21,22], there is an awakening to gain more insights on these non-*P. falciparum* species in order to develop improved malaria control measures. 

We have carried out a population genetic analyses of Poc and Pow from four countries that can be markedly grouped into Central Africa (Gabon); Horn of Africa (Ethiopia); East Africa (Kenya) and West Africa (Senegal), based on available sequence data in public databases. Since this is a comparative genetics study, Gabonese isolates were compared with Ethiopian isolates, while Kenya isolates were compared with Senegalese isolates. 

The diversity and average number of nucleotide difference (k) of Poc from Gabon (representing central Africa) was higher than that from Ethiopia. The two Ethiopian sequences retrieved show no difference between the nucleotides. In contrast, Pow from Ethiopia showed higher diversity in terms of nucleotides and haplotypes, ultimately resulting in a very high nucleotide difference. Although, previous studies have shown dimorphism in sexual genes of Poc and Pow [34] and marked differences between the diversity of other genes in Poc and Pow globally [3], in Bangladesh [35], and Thailand [36], none has evaluated the genetic diversity and phylogenetic relatedness of these subspecies in the continent with the highest burden of malaria. The difference in diversity observed between Poc and Pow from Ethiopia and Gabon probably reflects a pattern that has been seen amongst *P. falciparum* parasite population from Ethiopia, which has been shown to be uniquely different and diverse from *P. falciparum* parasite in different parts of sub-Saharan Africa [37]. The low genetic diversity observed between both subspecies and countries can possibly be attributed to low levels of recombination occurring in Poc and Pow. Nevertheless, that these subspecies showed observable difference in diversity poses another layer of challenge in the fight against malaria elimination, as non-*P. falciparum* malaria infections with their uniqueness would dominate post-elimination of *P. falciparum*.

With regards to comparison between Poc and Pow from Kenya representing East Africa and Senegal from West Africa, Poc and Pow from Kenya showed substantially high number of segregating sites and number of haplotypes in comparison to Senegal. However, the pairwise nucleotide diversity was much higher in Senegal than in Kenya, implying that though more haplotypes are recorded in Kenya, recombination events and transmission intensity are much higher in Senegal. This observation is in contrast to what has been known about parasite populations from East Africa, which together with those from the Horn of Africa, such as Ethiopia are uniquely different and more diverse [37] than those from other parts of Africa. One plausible explanation for this finding could be that the dominance of *P. falciparum* and *P. vivax* results in less competitive advantage of *P. ovale* and even *P. malariae*, and as such less recombination events. Nonetheless, the challenge that this place on malaria control strategy and intervention deployment needs to be critically evaluated.

Phylogenetic reconstruction of Poc and Pow sequences from Gabon and Ethiopia showed clear clustering of all Gabonese and Ethiopian isolates from each other for Poc. However, an Ethiopian isolate formed a unique clade with a Gabonese isolate with a bootstrap value of 86, denoting substantial level of similarity between the isolates. With regards to Poc and Pow from Kenya and Senegal, a reverse pattern was observed where an isolate from Kenya (Poc_4) was observed to form a clade with one from Senegal (Poc_1). However, Pow between these two countries show marked clustering within each country. These observations are not unexpected, though these countries are located in different parts of sub-Saharan Africa. The reason adduced for this observation is the constant migration of individuals along with the different pathogens they carry within the continent and parasite populations undergoing constant admixture. With such results, there is high potential that control interventions deployed in one country could be effective in the other since parasite from both countries shows some degree of relatedness.

Although, a more robust result from increased number of sequences could have provided better insights into the genetic events occurring between both subspecies in sub-Saharan Africa, nevertheless, these findings provide supporting evidence that can be strategically employed in the fight against non-*P. falciparum* malaria, where the uniqueness of parasite populations in each country needs to be considered when deploying control interventions.

## Figures and Tables

**Table 1:** Summary of sequences retrieved and utilized in this study.

Country	*P. ovale* subspecies	Target Gene	Number of Sequences	Reference	Accession Numbers
Gabon	*Po curtisi*	Small subunit ribosomal RNA	12	Groger and Fuehrer (unpublished)	MG847127-MG847138
	*Po wallikeri*	Small subunit ribosomal RNA	5	Groger and Fuehrer (unpublished)	MG847121-MG847126
Ethiopia	*Po curtisi*	Small subunit ribosomal RNA	2	[24]	KF536873—KF536874
	*Po wallikeri*	Small subunit ribosomal RNA	2	[24]	KF536875—KF536876
Kenya	*Po curtisi*	Tryptophan-rich protein—tra	4	[25]	KM494978, KM494979, KM494985, KM494986
	*Po wallikeri*	Tryptophan-rich protein—tra	5	[25]	KM494980—KM494984
Senegal	*Po curtisi*	Tryptophan-rich protein—tra	3	[26]	KX417701—KX417703
	*Po wallikeri*	Tryptophan-rich protein—tra	2	[26]	KX417699—KX417700

References relate to the studies that produced the sequences (incomplete) and sequence accession numbers are as indicated in the table. Note that Poc and Pow from Gabon are from unpublished work as indicated; tra—Tryptophan-rich protein. For Central African Republic, the cytochrome oxidase subunit 1 of Pow (*n* = 3) were the only sequences retrieved from databases, which were however excluded from this study (Table 1)

**Table 2:** Genetic diversity of *Po curtisi* and *Po wallikeri* in Gabon and Ethiopia using the small subunit ribosomal RNA gene.

	*Plasmodium ovale curtisi*		*Plasmodium ovale wallikeri*	
	Gabon	Ethiopia	Gabon	Ethiopia
Number of sequence isolates	12	2	5	2
Number of segregating (S)	6	0	0	3
Number of haplotypes	5	1	1	2
Haplotype diversity	0.667	0.000	0.000	1.0
Nucleotide diversity				
Θ_w_	0.00230	0.000	0.000	0.00426
Π	0.00359	0.000	0.000	0.00426
Average number of nucleotide differences (k)	1.273	0.000	0.000	3.0

No polymorphism seen in POC_Ethiopia and POW_Gabon sequence from Ethiopia, hence the other parameters were not computable. In order to use the DNA polymorphism program of the DnaSP software, the sequences must have at least one segregating (or polymorphic) site.

**Table 3:** Genetic diversity of *Po curtisi* and *Po wallikeri* in Kenya and Senegal using the tryptophan-rich antigen gene.

	*Plasmodium ovale curtisi*		*Plasmodium ovale wallikeri*	
	Kenya	Senegal	Kenya	Senegal
Number of sequence isolates	4	3	5	2
Number of segregating (S)	20	8	6	4
Number of haplotypes	4	3	5	2
Haplotype diversity	1.0	1.0	1.0	1.0
Nucleotide diversity				
Θ_w_	0.01451	0.02159	0.00455	0.01980
Π	0.01583	0.02159	0.00468	0.01980
Average number of nucleotide differences (k)	10	5.333	2.8	4.0

No polymorphism seen in POC_Kenya and POW_Senegal sequence from Ethiopia, hence the other parameters were not computable. In order to use the DNA polymorphism program of the DnaSP software, the sequences must have at least one segregating (or polymorphic) site.

## Data Availability

All data used in the works were obtained from published sequences and accessions numbers are provided in Table 1.
